# Revealing and mitigating the inhibitory effect of serotonin on HRP-mediated protein labelling

**DOI:** 10.1038/s41598-024-83928-w

**Published:** 2024-12-30

**Authors:** Zora Chui-Kuen Chan, Cheng Qi, Yuanhong Cai, Xin Li, Jing Ren

**Affiliations:** 1 Neurobiology Division, MRC Laboratory of Molecular Biology, Francis Crick Avenue, Cambridge, CB2 0QH UK; 2 Institute of Chemical Biology, Shenzhen Bay Laboratory, Shenzhen, 518132 China

**Keywords:** Biological techniques, Chemical biology, Neuroscience

## Abstract

Proximity-dependent biotinylation coupled with mass spectrometry enables the characterization of subcellular proteomes. This technique has significantly advanced neuroscience by revealing sub-synaptic protein networks, such as the synaptic cleft and post-synaptic density. Profiling proteins at this detailed level is essential for understanding the molecular mechanisms of neuronal connectivity and transmission. Despite its recent successful application to various neuronal types, proximity labelling has yet to be employed to study the serotonin system. In this study, we uncovered an unreported inhibitory mechanism of serotonin on horseradish peroxidase (HRP)-based biotinylation. Our result showed that serotonin significantly reduces biotinylation levels across various Biotin-XX-tyramide (BxxP) concentrations in HEK293T cells and primary neurons, whereas dopamine exerts minimal interference, highlighting the specificity of this inhibition. To counteract this inhibition, we demonstrated that Dz-PEG, an aryl diazonium compound that consumes serotonin through an azo-coupling reaction, restores biotinylation efficiency. Label-free quantitative proteomics confirmed that serotonin inhibits biotinylation, and that Dz-PEG effectively reverses this inhibition. These findings highlight the importance of accounting for neurotransmitter interference in proximity-dependent biotinylation studies, especially for cell-type specific profiling in neuroscience. Additionally, we provided a potential strategy to mitigate these challenges, thereby enhancing the accuracy and reliability of such studies.

## Introduction

Serotonin is a monoamine neurotransmitter synthesized from the amino acid tryptophan through a two-step enzymatic process: first, tryptophan is hydroxylated by tryptophan hydroxylase to form 5-hydroxytryptophan (5-HTP), which is then decarboxylated by aromatic L-amino acid decarboxylase to produce serotonin. The central serotonin system contains serotonin-producing neurons that are located at a few discrete nuclei in the brainstem and their extensive projections spread throughout the entire brain^1^. It powerfully modulates a broad range of brain functions, including mood, cognition, sleep, and etc^2^. After serotonin is released to the extracellular space, the serotonin transporter (SERT), which locates at the axonal terminal membrane, transports it back to the serotonin neuron, known as a process of serotonin reuptake. SERT is the most widely used pharmacological target for treating depression and anxiety^3,4^.

It has long been argued that the serotonin system uses both classical wired transmission and volume transmission. Ultrastructural characterization of mammalian serotonin axons reveals that there are both junctional and non-junctional varicosities^5^. Although both serotonin and dopamine are monoamine neuromodulators that utilize volume release, in comparison to the well-studied dopamine system, our understanding of the molecular mechanisms underlying serotonin transmission remains limited^6^. Critical questions remain unanswered: What is the subsynaptic protein network governing serotonin release? How are its postsynaptic targets specified? How are serotonin receptors precisely positioned? Addressing these questions is essential for developing a detailed molecular and cellular understanding of serotonin transmission. This knowledge will not only deepen our insights into how serotonin regulates brain functions but also help identify new targets for treating mental disorders such as depression and anxiety.

Comprehensive proteomic profiling of serotonergic terminal surfaces represents a crucial step toward achieving these goals. Proximity-dependent biotinylation, coupled with mass spectrometry-based quantitative proteomics, is a powerful approach for identifying and characterising proteins that are in close proximity to a protein of interest within and between living cells^7–11^. This method allows for the high-resolution mapping of protein interactions and local proteomes by labelling neighbouring proteins with biotin through the use of peroxidases or biotin ligase fused to specific proteins or subcellular regions. In peroxidase-mediated biotinylation, biotin-phenol derivatives are activated by horseradish peroxidase (HRP) or engineered ascorbate peroxidase (APEX) in the presence of H_2_O_2_^12,13^. These produces highly reactive biotin-phenoxyl radicals, which covalently bind to electron-rich amino acids such as tyrosine residues on nearby proteins, efficiently labelled proteins in close proximity to the peroxidase with biotin. BxxP is particularly advantageous for mapping extracellular surface proteins, offering a detailed view of the protein composition on cell membranes because BxxP is impermeable to cells^9^. The biotinylated proteins can then be isolated after cell lysis and characterised by mass spectrometry.

Proximity labelling has been proven invaluable in understanding signalling pathways in neuroscience. Researchers can elucidate the molecular architecture of neurons and synapses by tagging proteins near a target protein^14^. Recent applications of this technique have provided insights into the composition of sub-synaptic structures, including both the excitatory and inhibitory synaptic cleft in cultured mammalian neurons^9,15,16^. The method has also been applied in vivo to uncover the wiring regulators in *Drosophila*^17^. A comparison of cell-surface proteomes between developing and mature cerebellar Purkinje cells in mice using cell-type-specific proximity-dependent biotinylation has revealed regulators of dendritic morphogenesis^18^. Despite the broad influence of monoamine modulation, it is surprising that dopamine and serotonin receptors and transporters were absent from the list of proteins identified in Purkinje cells. This raises the question of whether serotonin- and dopamine-related proteins might be resistant to HRP-mediated proximity labelling.

It has been reported that serotonin can be oxidised and forms dimers in the presence of H_2_O_2_ and HRP^19^, while HRP can oxidise dopamine to generate phenoxyl radicals^20^. Both serotonin and dopamine form radicals catalysed by HRP in the presence of H_2_O_2_. Since peroxidase-mediated proximity labelling relies on generating phenoxyl radicals to attach to the electron-rich amino acid residues, it is crucial to investigate whether serotonin and dopamine interfere with this biotinylation process. This study explores the potential impact of serotonin and dopamine on the efficiency and specificity of peroxidase-mediated proximity labelling. Using a cell-based biotinylation assay, we showed that serotonin, rather than dopamine, inhibits both HRP and APEX-mediated biotinylation. In cultured primary neurons, we observed decreased biotinylation levels and a reduced biotinylation reaction radius in the presence of serotonin. To mitigate this effect, we synthesized an aryl diazonium compound, Dz-PEG, which consumes serotonin through an azo-coupling reaction. Further investigation confirmed that preincubation with Dz-PEG effectively counteracts the inhibitory effect of serotonin. Label-free quantitative proteomics demonstrated that serotonin significantly reduces the biotinylation of a broad range of proteins, with a notable impact on SERT. However, this reduction can be reversed by Dz-PEG.

## Results

### Serotonin inhibits peroxidase-mediated biotinylation

**Fig. 1:**
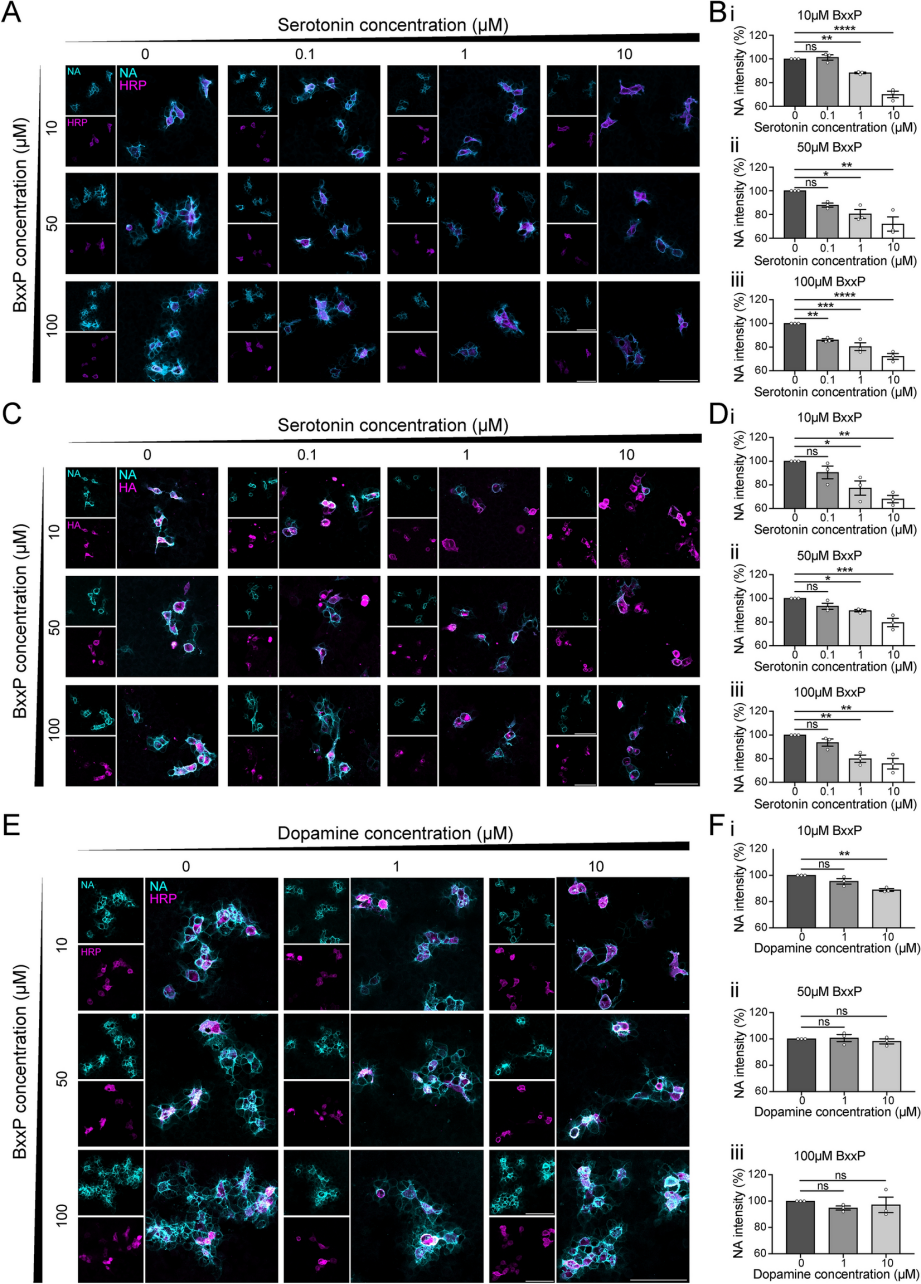
The effect of serotonin and dopamine on proximity labelling. (**A**) Representative images showing the effect of increasing serotonin concentrations on HRP-mediated biotinylation in HEK293T cells at varying BxxP concentrations. (**B**) Quantitative analysis of serotonin’s effect on HRP-mediated biotinylation at BxxP concentrations of (**i**) 10 µM, (**ii**) 50 µM, and (**iii**) 100 µM. Data are presented as the mean of n = 5 random views per serotonin concentration from three independent experiments. (**C**) Representative images showing the effect of increasing serotonin concentrations on APEX2-mediated biotinylation in HEK293T cells at varying BxxP concentrations. (**D**) Quantitative analysis of serotonin’s effect on APEX2-mediated biotinylation at BxxP concentrations of (**i**) 10 µM, (**ii**) 50 µM, and (**iii**) 100 µM. Data are presented as the mean of n = 5 random views per serotonin concentration from three independent experiments. (**E**) Representative images showing the effect of increasing dopamine concentrations on HRP-mediated biotinylation in HEK293T cells at varying BxxP concentrations. (**F**) Quantitative analysis of dopamine’s effect on HRP-mediated biotinylation at BxxP concentrations of (**i**) 10 µM, (**ii**) 50 µM, and (**iii**) 100 µM. Data are presented as the mean of n = 5 random views per serotonin concentration from three independent experiments. Scale bars represent 100 µm. Data are presented as mean ± SEM. One-way ANOVA with Dunnett’s multiple comparisons test, *, **, ***, **** represent *p* ≤ 0.05, 0.01, 0.001 and 0.0001 respectively. ns: non-significant.

To assess the impact of serotonin on proximity labelling, we evaluated the biotinylation efficiency at different extracellular serotonin concentrations. We expressed a PDGFR membrane-tagged HRP on the surface of HEK293T cells to label plasma membrane proteins by HRP-mediated reactions^8^. Serotonin was introduced alongside BxxP and H_2_O_2_. The use of surface HRP and BxxP enables the biotinylation of predominantly membrane proteins. Neutravidin (NA) is a deglycosylated form of avidin and has a strong affinity for biotin. Here we used NA staining to label biotinylated proteins and quantify the level of biotinylation. Across all BxxP concentrations (10, 50 and 100 µM), the level of biotinylation was inversely proportional to the concertation of serotonin (0.1, 1 and 10 µM) (Fig. 1A). In both conditions of 10 and 50 µM BxxP, either 1 or 10 µM serotonin significantly reduced biotinylation levels. At 1 µM serotonin, NA fluorescence intensity decreased by 13% and 20%, respectively; at 10 µM serotonin, it decreased by about 30% (Fig. 1B). In the condition of 100 µM BxxP, all serotonin concentrations applied significantly lowered biotinylation levels, with even 0.1 µM serotonin causing a 15% reduction. These results indicate that serotonin inhibits HRP-mediated biotinylation.

Like HRP, APEX also catalyses the oxidation of substrates such as biotin-phenol or biotin tyramide in the presence of H_2_O_2_. To further measure the effect of serotonin on APEX-mediated biotinylation, we expressed membrane-tagged APEX2 on the surface of HEK293T cells and performed biotinylation assays. Consistent with the HRP-mediated biotinylation results, 1 and 10 µM serotonin significantly decreased biotinylation levels across all BxxP concentrations (Fig. 1C, D). This demonstrates that serotonin inhibits both HRP- and APEX-mediated biotinylation.

Serotonin is a monoamine neurotransmitter that shares chemical similarities with other monoamines, such as dopamine and norepinephrine. To determine if dopamine exhibits a similar inhibitory effect as serotonin, we employed the same cell-based biotinylation assay used in assessing the impact of serotonin on proximity labelling. We expressed HRP on the surface of HEK293T cells and applied dopamine along with BxxP and H_2_O_2_. In contrast to serotonin, dopamine caused only a modest reduction in biotinylation levels (Fig. 1E, F). When 10 µM BxxP was applied, 10 µM dopamine induced an 11% decrease in biotinylation. No interference was observed in other conditions (Fig. 1F). These findings suggest that, despite its structural similarity to serotonin, dopamine only slightly inhibits peroxidase-mediated biotinylation.

### Azo-coupling reaction rescues HRP-mediated biotinylation from serotonin-induced inhibition

**Fig. 2:**
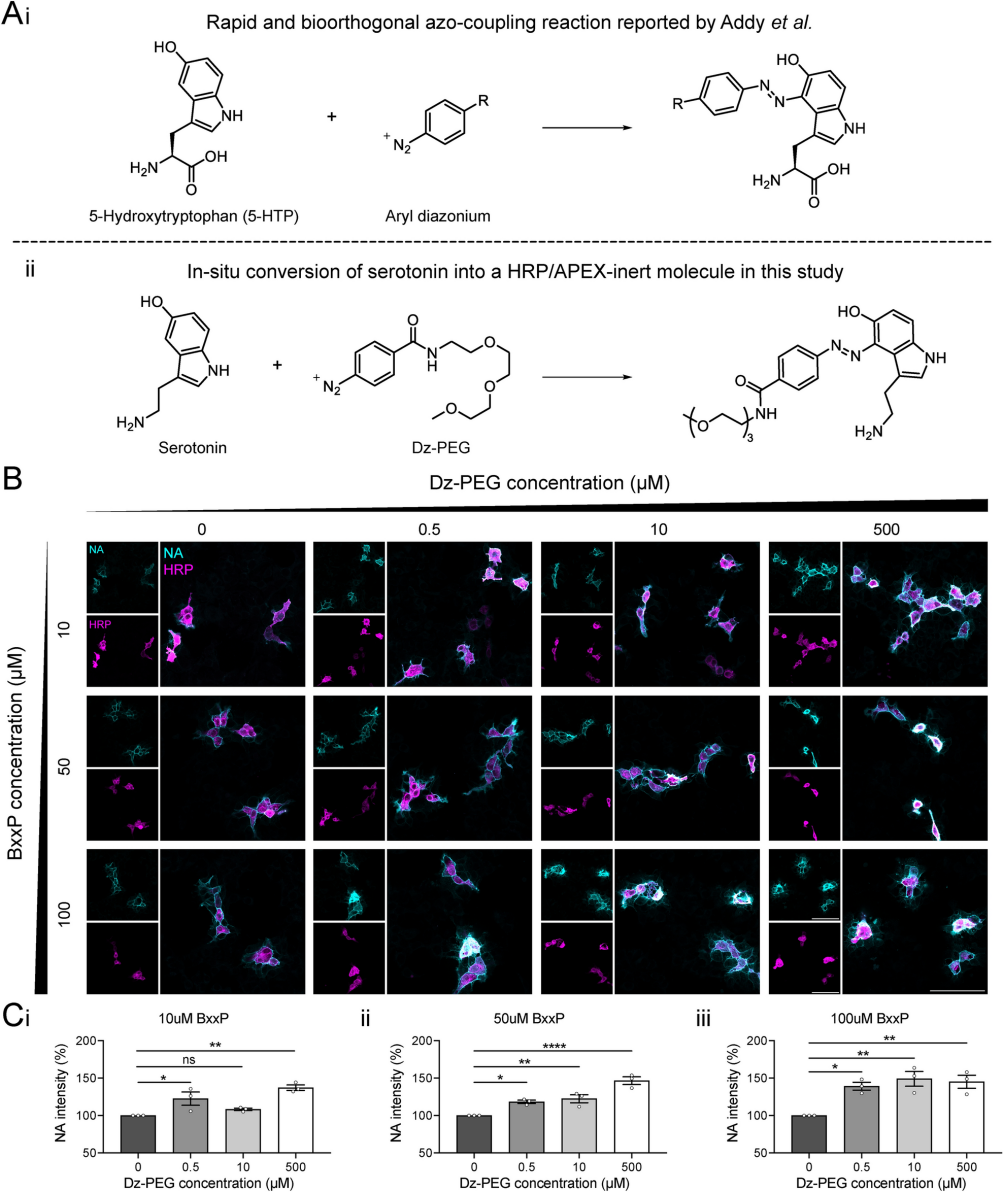
Dz-PEG rescues the inhibitory effect of serotonin on proximity labelling. (**A**) Azo-coupling reaction mediated by (**i**) Aryl diazonium, (**ii**) Dz-PEG. (**B**) Representative images showing the effect of preincubation with increasing Dz-PEG concentrations on the inhibition of HRP-mediated biotinylation by 10 µM serotonin in HEK293T cells at various BxxP concentrations. (**C**) Quantitative analysis of Dz-PEG’s effect on the inhibition of HRP-mediated biotinylation by 10 µM serotonin at BxxP concentrations of (**i**) 10 µM, (**ii**) 50 µM, (**iii**) 100 µM. Scale bars represent 100 µm. Data are presented as the mean of n = 5 random views per serotonin concentration from three independent experiments. Scale bars represent 100 µm. Data are presented as mean ± SEM. One-way ANOVA with Dunnett’s multiple comparisons test, *, **, **** represent *p* ≤ 0.05, 0.01 and 0.0001 respectively. ns: non-significant.

The inhibition brought by serotonin on peroxidase-mediated biotinylation will lead to biased or even failure on proteomic profiling after proximity labelling when applied in the serotonin system. Serotonin (i.e., 5-hydroxytryptamine) is a phenolic compound with a hydroxyl group attached to its benzene ring, which resembles the structure of biotin-phenol and may therefore competitively inhibit HRP/APEX-mediated biotinylation. A previous study has demonstrated that azo-coupling reactions between a series of aromatic diazonium ions and 5-hydroxytryptophan having fast reaction rates and good biorthogonality (Fig. 2Ai)^21^. We reasoned that the depletion of serotonin through in-situ conversion of the molecule into HRP/APEX-inert ones would rescue biotinylation (Fig. 2Aii). Considering the high structural similarity shared by serotonin and 5-hydroxytryptophan, we developed an aryl diazonium compound, Dz-PEG, and investigated whether this compound could alleviate the inhibition of serotonin on HRP-mediated biotinylation (Fig. 2).

We preincubated various concentrations of Dz-PEG with serotonin to compare the effect on the inhibition of biotinlyation against the serotonin-only group by using the NA staining assay on HEK293T cell. We used 10 µM serotonin, which had shown a significant inhibitory effect (Fig. 1B). Our results revealed that biotinylation levels increased as the concentration of Dz-PEG rose, across all BxxP concentrations (Fig. 2B, C). At 50 and 100 µM BxxP, all tested concentrations of Dz-PEG significantly relieved the inhibition effect of serotonin on biotinylation. Specifically, at 50 µM BxxP, NA fluorescence intensity increased by 18% to 47% as Dz-PEG concentrations rose from 0.5 to 500 µM. At 100 µM BxxP, approximately 39–49% increasement were observed with 0.5 to 500 µM Dz-PEG. These results demonstrate that Dz-PEG effectively consumes serotonin and counteracts its inhibitory effect on HRP-mediated biotinylation, potentially by blocking the hydroxyl group on serotonin and preventing radical formation.

### Serotonin decreases the biotinylation reaction radius in neurons

**Fig. 3:**
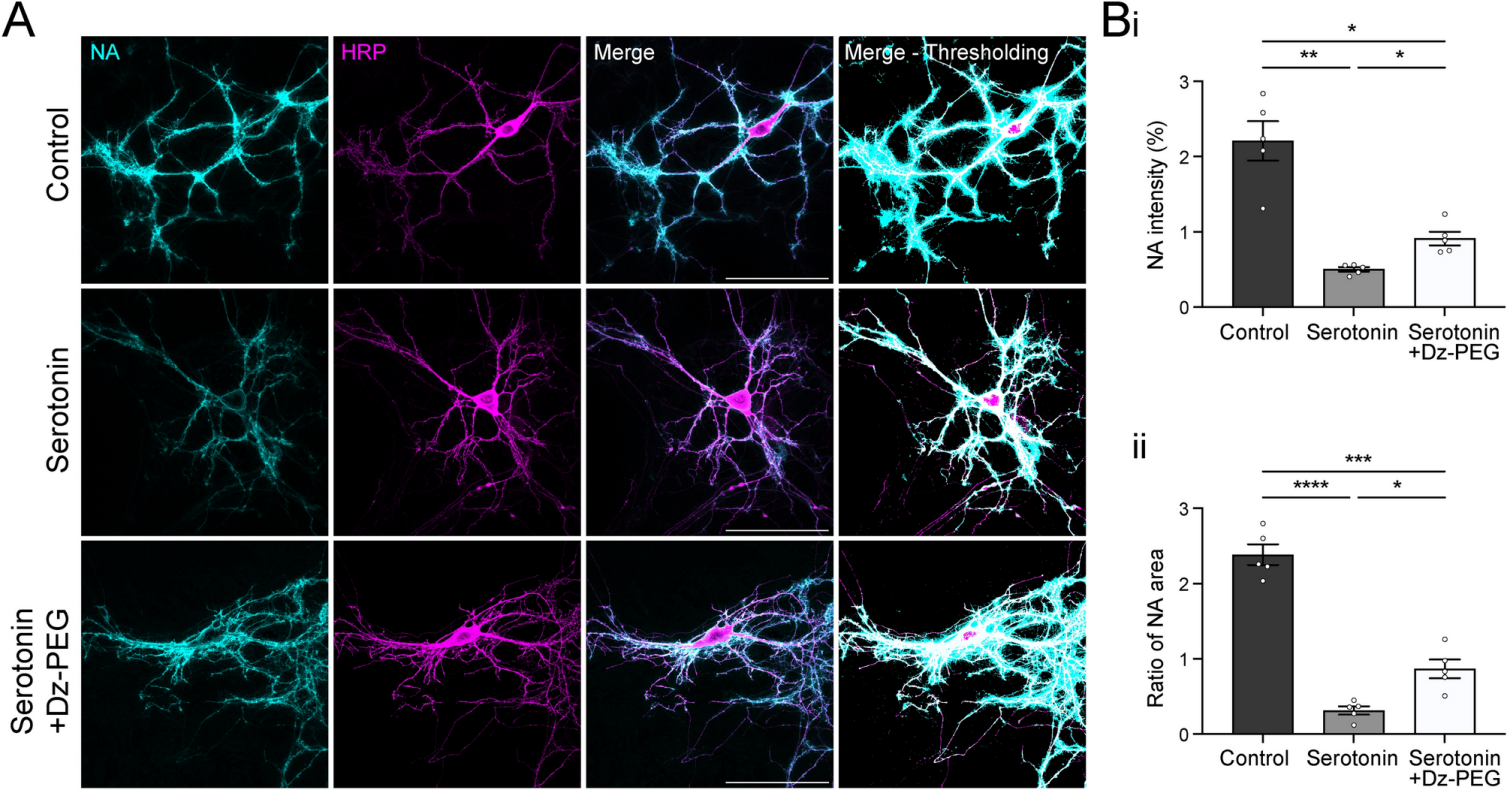
The effect of serotonin on the biotinylation reaction radius in HRP-expressing primary neurons, with and without Dz-PEG preincubation. (**A**) Representative images showing the effect of 10 µM serotonin alone and 10 µM serotonin with 500 µM Dz-PEG preincubation on biotinylation level in primary cortical neurons expressing HRP on the membrane. 8-bit pseudo-colour images represent the threshold used when quantifying the fluorescence intensity and area of HRP and NA for the result in **B**. (**B**) Quantification of the effects of 10 µM serotonin alone and 10 µM serotonin with 500 µM Dz-PEG preincubation on biotinylation level depicted by (**i**) fluorescence intensity of NA normalised by HRP expression, (**ii**) labelling area of NA normalised by HRP expression. Scale bars represent 100 µm. n = 5 neurons in each condition from one experiment. Data are presented as mean ± SD. One-way ANOVA with Dunnett’s multiple comparisons test, *, **, ***, **** represent *p* ≤ 0.05, 0.01, 0.001 and 0.0001 respectively.

Almost all brain regions receive input from serotonin neurons. The present of serotonin in the extracellular space of majority brain cells makes us wonder its influence on proximity labelling of neurons. To investigate if serotonin exerts similar effects on neurons, we expressed membrane-tagged HRP on primary cortical neurons and measured the biotinylation levels. HRP expression and NA staining are well overlapped from soma to neurites, indicating HRP-mediated biotinylation occurred throughout cellular surface of neurons (Fig. 3A). To account for the extensive membrane surface and variable morphology of neurons, we normalised the fluorescence intensity of NA to HRP levels for a more accurate assessment of biotinylation intensity. At 10 µM serotonin, the biotinylation intensity drastically decreased to 23%. This inhibitory effect was partially rescued to 41% by preincubating serotonin with 500 µM Dz-PEG before the reaction (Fig. 3Bi), which is consistent with previous results.

We observed that adding high levels of serotonin during HRP or APEX-mediated reactions on HEK293T cells also reduced the biotinylation area (Fig. 1A, C), and speculated that serotonin may decrease the effective reaction radius of biotinylation reaction. To validate this further, we quantified the NA area normalised to the HRP area in neurons. Serotonin at 10 µM significantly decreased the reaction radius to around 16%, while Dz-PEG reversed the reduction to 37% (Fig. 3Bii). This provides additional evidence of the inhibition of serotonin on HRP-mediated biotinylation and the rescue effect of Dz-PEG in primary neurons.

### Serotonin disrupts proximity labelling of high-affinity serotonin-binding proteins

Our findings revealed that serotonin inhibits HRP- and APEX-mediated biotinylation of proteins across varying extracellular serotonin concentration (Figs. 1, 2, 3). Based on these results, we hypothesize that in the neural system, proteins with high affinity for serotonin are challenging to be labelled by peroxidase-mediated biotinylation, due to their constant exposure to high serotonin levels. For instance, SERT, found on the plasma membrane of serotonergic neurons and responsible for serotonin reuptake, is constantly surrounded by high serotonin levels, making it difficult to be labelled by peroxidase-mediated biotinylation.

**Fig. 4:**
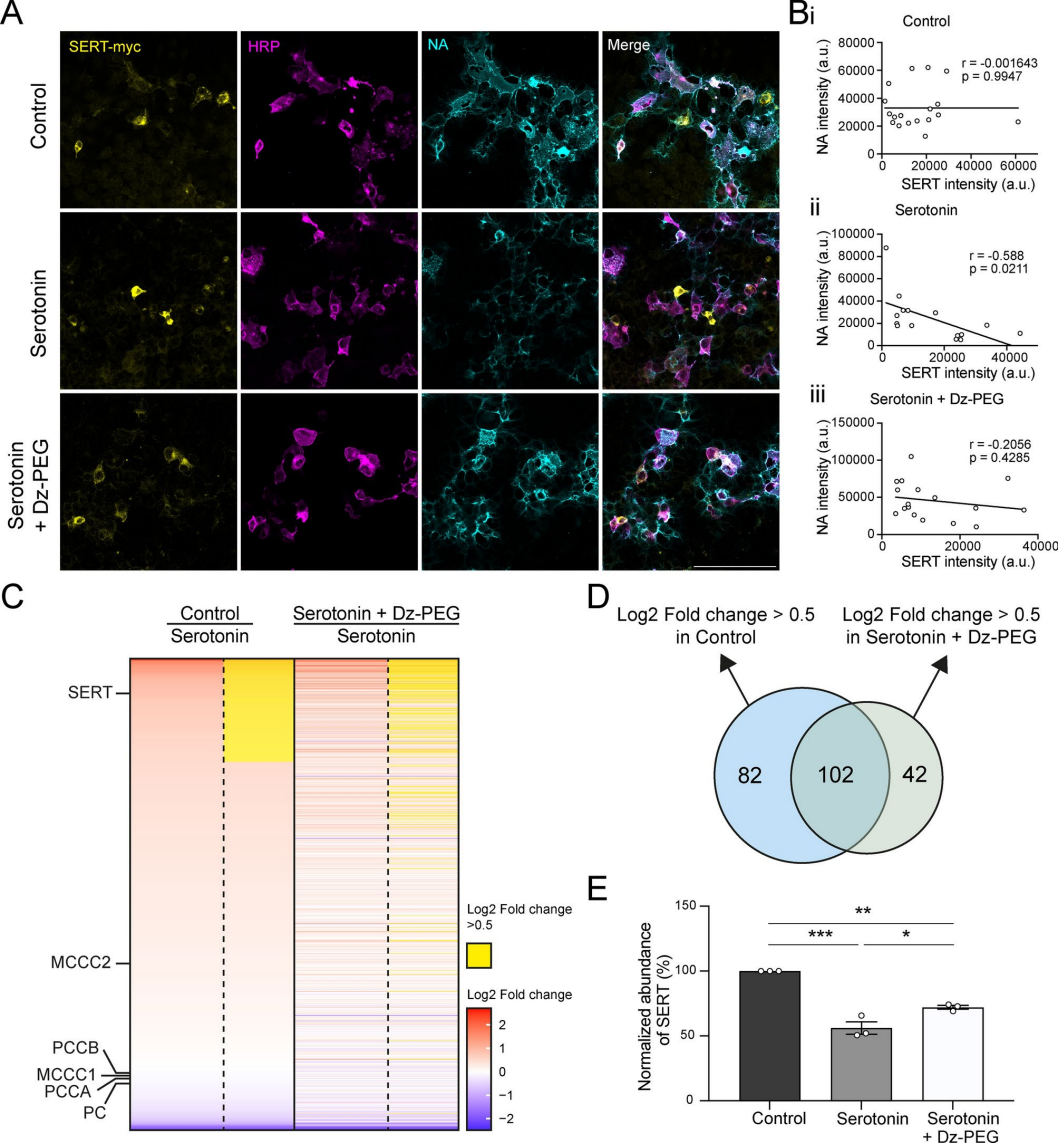
Serotonin broadly inhibits proximity labelling of proteins, with a significant impact on SERT. (**A**) Representative images showing the effect of 10 µM serotonin alone and 10 µM serotonin with 500 µM Dz-PEG preincubation on biotinylation levels in HEK293T cells co-expressing HRP and SERT on the membrane. (**B**) Scatter plot analysis showing the correlation between NA and SERT intensity in (**i**) control, (**ii**) serotonin-treated, and (**iii**) Dz-PEG preincubation groups. Pearson correlation coefficient (r) and p-values are shown for each condition. The black line indicates a linear correlation. n = 19 (control), 15 (serotonin), 17 (serotonin + Dz-PEG) cells. (**C**) Heatmap showing the Log2 fold change of proteins enriched in control and Dz-PEG preincubation groups compared to the serotonin-treated group. Endogenously biotinylated proteins, including MCCC2, PCCB, MCCC1, PCCA, and PC, were used for normalization. Yellow indicates proteins with a Log2 fold change over 0.5. (**D**) Venn diagram showing the number of proteins with a Log2 fold change > 0.5 in control group (blue) and Dz-PEG preincubation group (green) compared to serotonin-treated alone group. (**E**) Quantification of normalised SERT abundance in control, serotonin-treated, and Dz-PEG preincubation groups. Scale bars represent 100 µm. n = 3 replicates in each condition from one experiment. Data are presented as mean ± SEM. One-way ANOVA with Tukey’s multiple comparisons test, *, **, *** represent *p* ≤ 0.05, 0.01 and 0.001 respectively.

To test this hypothesis, we set out to test if SERT can be successfully enriched by proximity labelling with the present of serotonin. We co-expressed membrane-tagged HRP and SERT in HEK293T cells and performed biotinylation assays. We have three groups: no serotonin treatment, 10 µM serotonin added alone and 10 µM serotonin pre-incubated with 500 µM Dz-PEG (Fig. 4A). Interestingly, we found that in the serotonin-treated group, NA intensity was negatively correlated with SERT expression level. In contrast, there was no significant correlation between SERT expression and NA intensity in the group without serotonin or the one pre-incubated with Dz-PEG (Fig. 4B). This observation suggests that, due to its high affinity for serotonin, the expression of SERT compromises the biotinylation reaction of other surrounding proteins that were exposed to serotonin in the media.

Next, we tested if the inhibitory effect of serotonin on biotinylation will influence protein detection by mass spectrometry (MS) after proximity labelling and enrichment. Cell lysates from the three experimental groups were collected, and biotinylated proteins were enriched using streptavidin beads. A total of 1572 proteins were identified through label-free quantitative proteomic analysis (LFQ) (Supplementary Table 1). Among the three groups, the serotonin-treated group has the smallest number of biotinylated proteins, only 937 proteins were identified. Compared to the serotonin-treated group, 184 proteins exhibited more than a 0.5 log2 fold increase in abundance in the control group, and 102 of these proteins were restored to similar levels in the Dz-PEG pre-incubated group (Fig. 4C, D, Supplementary Table 1). Protein expression levels were normalised to ensure that the mean levels of endogenously biotinylated proteins (pyruvate carboxylase, 3-methylcrotonyl coA carboxylase and propionyl coA carboxylase) were consistent across the three groups. For proteins are quantified in all three groups, most exhibited lowest quantitative level in the serotonin-treated group (Fig. 4C). These results indicate that serotonin not only reduced the number of biotinylated proteins detectable by MS but also decreased the quantitative abundance of the ones identified.

Notably, compared to control group, SERT was significantly less abundant in serotonin-treated group, reduced by 50% (Fig. 4C, E). In Dz-PEG pre-incubated group, the abundance of SERT was rescued up to 83% (Fig. 4E). These findings indicate that the presence of serotonin inhibits HRP-mediated biotinylation of a large number of proteins, with SERT among one of the most affected proteins.

Collectively, our results suggest that proteomic profiling of subcellular structures using proximity labelling in environments are rich of serotonin is ineffective due to the inhibitory effect of serotonin. However, Dz-PEG has shown promise in counteracting this inhibition, enhancing the detection and profiling of proteins in neuronal structures or brain regions in close contact with serotonin.

## Discussion

The application of proximity labelling in neuroscience has significantly advanced our understanding of protein networks involved in neuronal connectivity, providing unprecedented insights into the proteomes within and between neurons. This approach extends beyond in vitro cultured neurons, having been successfully applied in vivo in both *Drosophila*^17,22,23^ and mice^18^. These successful applications also open promising new avenues for deciphering signals with cell type specificity. However, it is notable that serotonin or dopamine receptors and transporters were rarely detected in these studies, despite their known broad presence on the cell surface^18,24–26^. This discrepancy suggests the potential for serotonin and dopamine to interfere with peroxidase-mediated biotinylation, raising concerns about the use of proximity labelling for proteomic profiling in the monoamine neuronal systems.

Our study employed a cell-based biotinylation assay to investigate this potential interference. We found that serotonin exerts a strong inhibitory effect on both HRP- and APEX-mediated biotinylation, whereas dopamine’s effect is comparatively subtle. We proposed that serotonin radicals may compete with phenoxyl radicals for binding to electron-rich amino acids, thereby causing this inhibition^19^. In this study, we evaluated serotonin’s effects at concentrations ranging from 0.1 to 10 µM, and all three concentrations showed an inhibitory effect when 100 µM BxxP was applied (Fig. 1). Under physiological conditions in the brain, local extracellular serotonin concentrations can reach 100 nM during a single neuronal impulse and may rise to micromolar levels with repeated firing^27^. Additionally, serotonin concentrations within presynaptic vesicles can reach several hundred millimolar^28^, making the proteins distributed at the local releasing sites on the serotonin axonal terminals exposed in millimolar levels of serotonin. Consequently, using biotinylation based proximity labelling to profile protein networks in serotonin neurons from fresh brain tissues, as outlined in recent protocols^18^, is unlikely to be effective. Indeed, our data show that serotonin broadly inhibits biotinylation, reducing both the identification and quantification of proteins in proximity labelling. This effect notably impacts SERT, which is located along the serotonin-releasing sites in the brain. Interestingly, we also found that there were 22 proteins, including three histone proteins, that were significantly enriched in the serotonin-treated group comparing to the control group (Supplementary Table 1). Gene Ontology (GO) analysis revealed that these proteins are predominantly associated with “nucleosome assembly”, “nucleosome organization” and “chromatin assembly” (data not shown). Given that serotonylation of histone proteins is catalyzed by transglutaminase 2 (TGM2)^29^, we hypothesize that enhanced serotonylation, particularly in proteins linked to chromatin-related processes, may underlie the observed increase in biotinylation. These findings require further investigation into the interplay between HRP, serotonin, biotin phenol, and TGM2 to elucidate the molecular mechanisms driving this phenomenon.

Our results also suggest that proteins with a high affinity for serotonin, or those in serotonin-rich environments, may be less likely to be biotinylated and identified. This could introduce biases in proximity labelling-facilitated proteomics. To address the challenge of profiling the serotonin system by proximity labelling and minimize biases caused by the inhibitory effect of serotonin in other neuronal systems, we set out to find a way to consume serotonin in the extracellular space. We found that the azo-coupling reaction, which occurs between a diazonium compound and another aromatic compound to produces an azo compound^30^, successfully rescues the inhibition caused by serotonin on HRP-mediated biotinylation (Figs. 2, 3, 4). We synthesized Dz-PEG, who can restore the detection of biotinylated proteins that were disrupted by serotonin. The proteins rescued by Dz-PEG present an intriguing interplay between expected and unexpected outcomes, highlighting the complexity of biotinylation mediated by HRP. Proteins such as FAM168A, FAM168B, CD59, and ASM3B, which contain more than 15% electron-rich amino acids (tyrosine, tryptophan, histidine, cysteine, and methionine), were anticipated to be effectively rescued due to the abundance of potential biotinylation sites. However, unexpectedly, proteins like SRRM2 and MDC1, which have less than 5% of these amino acids, were also rescued, suggesting that the Dz-PEG effect is not only dependent on amino acid composition. This observation points to the importance of structural context, particularly the surface accessibility of electron-rich residues, in determining biotinylation efficiency. Therefore, the rescue phenomenon appears to involve not only the biochemical availability of reactive amino acids but also the conformational exposure and dynamic folding of proteins, which may influence residue accessibility for biotinylation.

The successful application of azo-coupling reaction may also explain why dopamine’s inhibitory effect is much weaker compared to serotonin. Dopamine has two adjacent hydroxyl groups on its benzene ring, unlike serotonin, which has only one. This difference could cause intramolecular interference in dopamine, which may prevent effective oxidation by peroxidase. We propose that norepinephrine, another monoamine neurotransmitter with two adjacent hydroxyl groups, may also have a limited inhibitory effect on peroxidase-based biotinylation. However, the co-existence of serotonin, dopamine, and norepinephrine in the extracellular space could produce combined effects on biotinylation under physiological conditions in the brain. Given the extremely high local concentrations at the releasing sites, further investigation is necessary before applying proximity labelling to the monoamine systems.

Our findings provide a valuable reference for future studies aiming to profile surface proteins of serotonergic neurons. Identifying Dz-PEG as an agent that mitigates serotonin’s inhibitory effects paves the way for comprehensively profiling the proteome of the serotonin system. This advancement also suggests potential improvements in proximity labelling methods for various neural systems, particularly those significantly influenced by monoamine transmitters.

## Methods

### Cell cultures

The HEK293T cell line was purchased from ATCC (ATCC, CRL-3216). The HEK293T cell line was cultured in DMEM (Gibco, 31966047) supplemented with 10% fetal bovine serum (FBS, Gibco, 10270–106) and 1% penicillin/streptomycin (Gibco, 15140122) at 37 °C under 5% CO2. Cells were passaged at 80%–90% confluence by trypsinisation and reseeded.

### Primary neuronal culture

Cortical neurons were prepared from male and female postnatal day P0 mouse pups. P0 pups were first killed by Schedule 1 method (decapitation), and cortical tissue was collected and dissociated using 0.25% Trypsin and DNase I (NEB, M0303AA) in dissociation medium, then plated onto 0.17 mm thick polymer coverslips in 24-well plates (Ibidi, 82426) in plating medium and cultured at 37 °C under 5% CO_2_ for 4 h before switching to maintenance medium. Dissociation medium consisted of Ca^2+^ and Mg^2+^-free HBSS supplemented with 1 mM sodium pyruvate (Invitrogen, 11360070), 0.1% glucose (Sigma-Aldrich, G-6152), and 10 mM HEPES [pH 7.3] (Sigma-Aldrich, H-4034). Plating medium was Basal Medium Eagle (Invitrogen, 21010046) supplemented with 10% FBS (Gibco, 16140071), 0.45% glucose, 1 mM sodium pyruvate, 2 mM L-glutamine (Invitrogen, 25030081), and 1% (v/v) penicillin/streptomycin (Gibco, 15140122). Maintenance medium was Neurobasal medium (Invitrogen, 21103049) supplemented with 2% (v/v) B27 (Invitrogen, 17504044), 2 mM L-glutamine, and 1% (v/v) penicillin/streptomycin. Polymer coverslips in 24-well plates were pre-incubated at room temperature with 0.1 mg/mL poly-d-lysine (PDL, Gibco, A3890401) followed by 1 mg/mL mouse laminin (Sigma-Aldrich, L2020) in water. Maintenance medium was refreshed every 3 days.

All experiments related to the use of mice in the Medical Research Council Laboratory of Molecular Biology were carried out in accordance with the UK Animals (Scientific Procedures) Act of 1986, with local ethical approval provided by the Medical Research Council Laboratory of Molecular Biology Animal Welfare Ethical Review Board and overseen institutionally by designated animal welfare officers (Animal Project License PP6471806), and are in accordance with the ARRIVE guidelines.

### Plasmids and cloning

Plasmids were obtained from Addgene as follows:

- pCAG-HTP-TM (#44441)
- pcDNA3-APEX2-NES (#49386)
- pCEP4-SERT-myc (#107456)

Plasmids created in this study:

- pCAG-APEX2-TM

Gibson assembly was used for cloning all plasmids created in this study. The pCAG-HTP-TM plasmid was digested with AgeI and HindIII restriction enzymes to remove HRP-TM, and APEX2 was amplified with primers overlapping with the digested vector.

### Cell-based biotinylation assays

**Scheme 1.**
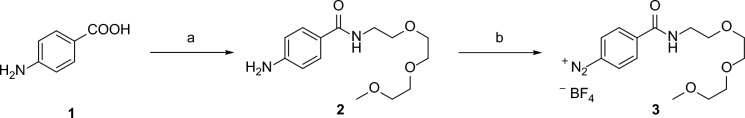
Synthetic route of Dz-PEG. Conditions: (**a**) CH_3_(OCH_2_)_3_NH_2_, HOBt, EDC, DMF; (**b**) **i**. NaNO_2_, 6 M HCl; **ii**. 48% HBF_4_.

HEK293T cells were seeded in 6-well plates at 1 × 10^6^ cells per well and transfected after 24 h with a recombinant HRP plasmid (2 μg pCAG-TM-HRP) using PEI (Polysciences, 24765) at approximately 80% confluency. Following overnight transfection, HEK293T cells were plated on PDL-coated 24-well polymer bottom plates (Ibidi, 82,426) and allowed to grow for 24 h. Cells were then live-labelled with 100 µM BxxP (ApexBio, A8012) and 0.003% H_2_O_2_ in PBS for 2 min at room temperature. The reaction was quenched immediately by replacing the medium with PBS (quenching buffer) containing 10 mM sodium azide, 10 mM sodium ascorbate, and 5 mM Trolox. Cells were washed twice more with the quenching buffer. For biochemical characterisation or proteomic sample collection, cells were snap-frozen in liquid nitrogen and stored at − 80 °C. For immunocytochemistry, cells were fixed and stained as described below (Scheme 1).

### Synthesis of Dz-PEG

^1^H NMR spectra were recorded on Bruker 400 or 600 MHz spectrometer. Chemical shifts were reported in values (ppm), and *J* values were reported in Hertz. Reagents and solvents were purchased from common commercial suppliers and were used without further purification.

Synthesis of Compound **2**: 4-Aminobenzoic acid (**1**) (137 mg, 1.0 mmol, 1 equiv.), HOBt (216 mg, 1.6 mmol, 1.6 equiv.), and EDC (300 mg, 1.6 mmol, 1.6 equiv.) were dissolved in DMF (4 mL). To this solution CH_3_(OCH_2_)_3_NH_2_ (260 mg, 1.6 mmol, 1.6 equiv) was added and the mixture was stirred overnight. The reaction was quenched by adding 20 mL water and extracted with ethyl acetate (3 × 20 mL). The combined organic phase was dried over Na_2_SO_4_ and concentrated by rotovap. The crude product was purified by column chromatography to give 174 mg of colourless solid **2**. Yield 62%. ^1^H NMR (600 MHz, DMSO-*d*_6_) δ 7.99 (t, *J* = 5.6 Hz, 1H), 7.55 (d, *J* = 8.3 Hz, 2H), 6.53 (d, *J* = 8.2 Hz, 2H), 5.58 (s, 2H), 3.53 – 3.46 (m, 8H), 3.41 (dd, *J* = 5.6, 3.9 Hz, 2H), 3.35 (d, *J* = 5.9 Hz, 2H), 3.22 (s, 3H); ^13^C NMR (151 MHz, DMSO) δ 166.76, 152.02, 129.13, 121.60, 112.97, 71.73, 70.18, 70.07, 70.05, 69.64, 58.51, 39.38.

Synthesis of Compound **3**: Compound **2** (320 mg, 1.1 mmol, 1 equiv.) was dissolved in 6 M HCl (1 mL) and incubated on ice for 15 min. To this solution NaNO_2_ (97 mg, 1.4 mmol, 1.2 equiv.) was added and stirred for 30 min on ice. 1.6 mL 48% HBF4 was added and kept stirring for 30 min on ice. The generated precipitate was filtered and dried in vacuum to give 87 mg Compound **3** as tetrafluoroborate salt of Dz-PEG. Yield 21%; ^1^H NMR (600 MHz, Methanol-*d*_4_) δ 8.74 (d, *J* = 8.7 Hz, 2H), 8.33 (d, *J* = 8.8 Hz, 2H), 3.70 (t, *J* = 5.3 Hz, 2H), 3.67 (s, 4H), 3.64 (q, *J* = 5.6, 5.0 Hz, 4H), 3.57–3.53 (m, 2H), 3.35 (s, 3H); ^13^C NMR (151 MHz, Methanol) δ 164.95, 145.67, 132.60, 129.87, 117.43, 71.47, 70.14, 69.92, 69.84, 68.76, 57.65, 40.03.

### Serotonin, dopamine and Dz-PEG treatment

For experiments studying the effects of serotonin on HRP- or APEX2-mediated biotinylation in HEK293T cells (Fig. 1A to D), 0.1, 1, and 10 µM serotonin hydrochloride (Sigma, H9523-100MG) were mixed with 10, 50, and 100 µM BxxP, respectively, in culture medium containing 0.003% H_2_O_2_ before adding to cells. For experiments studying the effects of dopamine on HRP-mediated biotinylation in HEK293T cells (Fig. 1E, F), 1 and 10 µM dopamine hydrochloride (Sigma, H8502-5G) were mixed with 10, 50, and 100 µM BxxP, respectively, in culture medium containing 0.003% H_2_O_2_ before adding to cells. For experiments studying the effects of Dz-PEG on rescuing serotonin inhibition on HRP-mediated biotinylation in HEK293T cells (Fig. 2), 0.5, 10, and 500 µM Dz-PEG were mixed with 10 µM serotonin hydrochloride before combining with culture medium containing BxxP and H_2_O_2_. For experiments studying the effects of serotonin on HRP-mediated biotinylation in primary neurons and in HEK293T cells for label-free quantification (Figs. 3, 4), 10 µM serotonin hydrochloride were mixed with 500 µM Dz-PEG before combining with culture medium containing BxxP and H_2_O_2_.

### Lysis of HEK293T cells and biotinylated protein enrichment

Cells from two wells of a 6-well plate were harvested by scraping in 1.2 mL of lysis buffer (50 mM Tris–HCl [pH 8.0], 150 mM NaCl, 0.2% SDS, 0.5% sodium deoxycholate, 1% Triton X-100, 1 × protease inhibitor cocktail [Sigma-Aldrich, P8849], and 1 mM phenylmethylsulfonyl fluoride). Samples were briefly vortexed, followed by two rounds of 10 s sonication. Samples were rotated for 1 h at 4 °C and cleared by centrifugation at 16,000 × g for 15 min at 4 °C. Protein concentration was determined by BCA assay. Streptavidin magnetic bead slurry (Pierce, 88816), washed twice with RIPA lysis buffer, was added to 2000 µg protein in each sample and incubated overnight at 4 °C with gentle rotation. Beads were washed sequentially with RIPA lysis buffer, 1 M KCl, 0.1 M Na_2_CO_3_, and 2 M urea in 10 mM Tris–HCl [pH 8.0].

### On-beads digestion of biotinylated proteins

Proteins on beads were suspended in 2 mM DTT, 1.8 M urea and sequencing grade trypsin (Promega) was added to a final concentration of 5 ng/µL. After incubated for 3 h at 25 °C, supernatants were transferred to new eppendorf tubes. Beads were washed sequentially once with 2 M and once with 1 M urea buffer. The washes were combined with the corresponding supernatants and were then alkylated with 4 mM iodoacetamide (IAA) in the dark at 25 °C for 30 min. An additional 0.1 µg of trypsin (Promega) was added to the samples and incubated over night at 25 °C. Samples were acidified to 0.5% FA, and centrifuged at 18,000 × g for 10 min. Supernatants were desalted using custom-made C18 stage tips (3 M Empore) packed with Poros oligo R3 resin (Thermo Scientific). Stage tips were equilibrated with 80% acetonitrile (MeCN)/0.5%FA followed by 0.5%FA. Peptide mixtures were loaded onto stage tips and washed with 0.5% FA. Bound peptides were eluted stepwise with 30, 50 and 80% MeCN in 0.5% FA, and partially dried down in Speed Vac (Savant) before LC–MS analysis.

### Liquid chromatography with tandem mass spectrometry

LC–MS analyses were performed using a Q Exactive Plus hybrid quadrupole-Orbitrap mass spectrometer (Thermo Fisher Scientific) coupled to an automated Ultimate 3000 RSLC nano System, fitted with a PepMap Neo C18 5 μm 0.3X5 mm nano trap column (Thermo Fisher Scientific) and an Aurora Ultimate TS 75 μm × 25 cm × 1.7 μm C18 column (IonOpticks). Peptides were separated using buffer A (0.1% FA) and buffer B (80% MeCN, 0.1% FA) at flow rated of 300 nL/min and column temperature of 40 ⁰C. The mass spectrometer was operated in DDA mode, performed full-scan MS1, at m/z = 380–1500 with a resolution of 70 K, followed by MS2 acquisitions of the 15 most intense ions with a resolution of 35 K. NCE of 27% and isolation window = 1.2 m/z were used. Dynamic exclusion was set for 30 s.

### Mass spectrometry data processing

The raw data from LC–MS/MS were processed using MaxQuant (Cox and Mann) with the integrated Andromeda search engine (v.2.4.2.0). Data were searched against UniProt UP000005640_9606_human_proteome database (downloaded on 030523), in Label Free quantification mode. Carbamidomethylation of cysteines was set as fixed modification, while methionine oxidation and protein N-terminal acetylation were set as variable modifications. Protein quantification requirements were set at 1 unique and razor peptide. In the identification tap, second peptides and match between runs were not selected. Other parameters in MaxQuant were kept as default values.

### Proteomic data analysis with perseus

The MaxQuant output file, proteinGroups.txt, was processed using Perseus software (v2.0.10.0). After uploading the matrix, the data was filtered to remove identifications from the reverse database, identifications with only modified peptides, and common contaminants. The experiment was performed in triplicate for each treatment. Proteins with fewer than two unique peptides were filtered out. LFQ intensities were normalised using the average intensities of endogenous biotinylated proteins, including MCCC2, PCCB, MCCC1, PCCA, and PC. The triplicates were grouped based on experimental conditions. Only proteins with an LFQ value in all replicates across all conditions were retained for analysis, which was used to generate the heatmap and Venn diagram shown in Fig. 4C and D. The heatmap was generated by plotting the Log2 fold change, comparing the control or serotonin + Dz-PEG to serotonin-treated groups in R. A protein was considered enriched if it had a Log2 fold change greater than 0.5 compared to the serotonin-treated group and was visualized in the Venn diagram. To quantify SERT abundance, normalised LFQ intensities in the serotonin-treated and serotonin + Dz-PEG-treated groups were compared to the control.

### Fixation and immunostaining of cells

HEK293T cells were fixed with 4% paraformaldehyde in PBS at room temperature for 10 min, washed three times with PBS, and stained with NA (Invitrogen, 22832 and 84607) at 1:1000 dilution for 1 h to visualise the BxxP labelling site. After washing, cells were fixed again with 4% paraformaldehyde in PBS, permeabilised with 0.1% Triton X-100 in PBS for 15 min at room temperature, blocked with 3% BSA in PBS for 1 h, and incubated with primary antibodies diluted in 3% BSA in PBS (1:2500 goat anti-HRP [ImmunoReagents, GtxOt-096-D]; 1:1000 rabbit anti-HA [Cell Signaling Technology, 14697S]; 1:1000 rabbit anti-myc [Cell Signaling Technology, 2278S]) for 2 h at room temperature. After washing, cells were incubated with secondary antibodies (1:1000 Donkey anti-goat AlexaFluor 546 [Invitrogen, A11056], Donkey anti-goat AlexaFluor 647 [Invitrogen, A21447], Donkey anti-rabbit AlexaFluor 546 [Invitrogen, A10040]) in 3% BSA in PBS for 1 h. Following final washes with PBS, cells were stored in PBS with 0.02% sodium azide until imaged by fluorescence microscopy.

### Fluorescent imaging of biotinylation

Fluorescence images of HEK293T cells and primary neurons were acquired via confocal microscopy on a Zeiss inverted microscope (LSM 900) equipped with Zen software. 20 × and 40 × objectives were used for image acquisition, with Z-stack images acquired at a step size of 0.66 µm and 0.29 µm, respectively. Excitation wavelengths of 488 nm, 561 nm and 647 nm were used, and emission was collected in the 500–550 nm, 570–620 nm and 660–710 nm, respectively. Images were acquired at a resolution of 1024 × 1024 pixels or 2048 × 2048 pixels with a pinhole size of 1 Airy unit. Acquisition settings (laser intensity and gain) were standardised across experimental groups within each experiment. Image processing and analysis were performed using ImageJ (NIH).

### Quantification and statistical analyses

To quantify the normalised intensity of NA on HEK293T cells, background intensity was first averaged from selected background regions (18 to 24 images per experiment) using the Macros function in ImageJ (NIH). A 10-time background intensity threshold was set, and signals above the threshold were measured using the Macros function in Plugins. The average intensity shown in Figs. 1B, D, F, and 2B was calculated with the following function:

$$Average\, Intensity=\Sigma\, Integrated \,Intensity/\Sigma \,Area$$

To quantify the biotinylation intensity in primary cortical neurons, the threshold was first set using 5 times the background intensity, and signals above the threshold were measured. The measured fluorescence intensity of NA was then normalised to the HRP intensity in each neuron.

The same threshold was used to quantify the biotinylation reaction radius in primary cortical neurons as in quantifying biotinylation intensity. The normalised ratio of NA to HRP area was calculated using the following equation:

$$Normalized\, ratio\, of \,neutravidin \,to \,HRP \,area=\frac{Neutravidin \,area-HRP \,area}{HRP \,area}$$

In all figures, mean and SEM values were shown in the graphs unless otherwise specified. The numbers of biological replications and the statistical tests applied were specified in the figure legends.

## Supplementary Information

Supplementary Information.

## Data Availability

Data underlying the results presented in this paper are not publicly available at this time but may be obtained from the corresponding authors upon reasonable request.
